# Revealing associations between exposure to cyanobacteria toxins and COVID-19 outcomes in Colorado

**DOI:** 10.1101/2025.08.01.25332749

**Published:** 2025-08-05

**Authors:** Anne E Thessen, Shawn T O’Neil, Melissa A Haendel

**Affiliations:** 1 University of North Carolina Chapel Hill, Department of Genetics, Chapel Hill, North Carolina, USA

## Abstract

While environmental exposures are known to play a significant role in human disease, these effects are understudied compared to genomic and molecular components of disease. One example is the effect of chronic, low-level exposures to cyanobacteria toxins on health outcomes. Here we perform a retrospective analysis on real-world data in the National Clinical Cohort Collaborative (N3C) COVID Enclave, examining the possible impact of chronic exposure to cyanobacteria toxin on the severity of COVID-19 outcomes in patients from Colorado. We combined data from N3C, satellite data from the USEPA CyAN project, and field observations from the Colorado Department of Public Health & Environment (CDPHE). Our results show that COVID-19 patients living near recurring cyanobacteria blooms had 2.75 times higher odds of experiencing severe outcomes (hospitalization or death) than individuals who do not. In addition, living in a county with low to middle levels of poverty had protective effects. Further work is needed to understand the precise mechanism of action and fully understand the long-term risk of chronic exposures to low-level cyanobacteria toxins on health outcomes.

International Committee of Medical Journal Editors (ICMJE) Statement: Authorship was determined using ICMJE recommendations.

IRB: 24-2680

DUR ID: DUR-15EB88A

## Introduction

Many advances have been made in understanding genomic and molecular components of disease. The environmental component of disease is less well understood, but is known to play a significant role in cardiac, neurological, and immunological disease [1–3]. The effects of environmental algal toxins are not well understood, despite their impact on public health. In 2020, there were 227 harmful algal blooms reported in the US, resulting in 95 human and 1170 animal illnesses [4]. Most of these occurred in freshwater which had detectable amounts of microcystin, a toxin produced by a type of algae called cyanobacteria [4]. Toxin-producing cyanobacteria blooms are increasing globally and have caused many illnesses and deaths in humans and animals through an array of secondary metabolites that have hepatotoxic, neurotoxic, and skin-irritant properties [5,6]. Highly-visible acute mortality and illness events have been studied using field reports and laboratory investigations, but less is known about the public health consequences of repeated, low-level exposures to cyanobacterial toxins over long periods of time. This is partially due to their “silent” nature, but also due to the complexity of combining environmental and clinical data to investigate exposures of this type. Here, we combined clinical data from the National Clinical Cohort Collaborative (N3C) COVID Enclave [7] with water quality data from the USEPA CyAN [8] project to identify possible consequences of chronic cyanobacteria toxin exposure on COVID-19 outcomes.

The prevalence of toxin-producing cyanobacteria blooms in freshwater, including water used for drinking and recreation, has been increasing, caused by a combination of eutrophication and climate change [5,9,10]. The first documented deaths due to cyanobacteria toxins occurred in 1878 and involved cattle and sheep [11]. The first human intoxication event was recorded in 1931 when about 9,000 people developed acute gastroenteritis from cyanobacteria-contaminated drinking water [12]. Since that time, other reported symptoms in humans include skin rashes, skin and hepatic lesions, vomiting, headache, seizures, severe pneumonia, and death depending on the severity and mode of exposure. Several toxic events (resulting in death or illness) caused by cyanobacteria have been documented in multiple species, and it is well known that cyanobacteria produce hepatotoxins, neurotoxins, and skin irritants [6] (Table 1). Cyanobacteria toxins can be inhaled as an aerosol [13,14] and ingested in water or food [10]. The presence of cyanobacteria and their toxins in bioaerosols has been well documented [15,16], and exposure to toxin-containing aerosols may not be restricted to areas near an affected water body [17]. A previous study in California suggested that while proximity to a cyanobacteria bloom resulted in inhaling aerosolized cyanobacteria toxins, it was unlikely to cause adverse acute effects in healthy individuals over the short term [13]. Laboratory studies demonstrated that cyanobacteria aerosols can cause irritation and inflammatory injuries in human upper respiratory airway epithelial cells [18]. A study examining cyanobacteria in the human respiratory tract found no relationship between the presence of cyanobacteria in a patient and that patient’s proximity to a water body [17]. Field data (*in situ* samplers) suggest that cyanobacteria toxins can travel over a mile from source [19] and can be found in indoor environments [15]. Controlled studies show that cyanobacteria toxins quickly decay in sunlight [20] and models suggest that cyanobacteria toxins are only able to travel approximately half a mile from source before decaying [21].

Less understood is the relationship between chronic disease and repeated exposure to cyanobacteria toxins at levels too low to cause acute illness. Repeated exposure to low-levels of cyanobacteria toxins may occur via food, absorption through the skin, or inhalation. Several epidemiological studies have found a link between the presence of cyanobacteria toxins in drinking water and liver cancer around the world [22–25]. Increasing evidence suggests that environmental toxins play an important role in liver cancer development and could be contributing to the increase in chronic liver disease [26]. Several studies suggest that aerosolized cyanobacteria toxins can be inhaled [27] and it has been hypothesized that exposure through inhalation or ingestion is connected to neurodegenerative disease [28–31]. In addition to being directly harmful, the tissue damage caused by cyanobacteria toxins could potentially increase susceptibility to other diseases, including COVID-19. Studies have shown that patients with chronic respiratory, kidney, liver, and neurological disease were at higher risk of hospitalization due to COVID-19 [27]. Exact amounts of toxin that result in organ damage, and lag time between exposure and organ damage without acute illness in humans is unknown.

Climate change, in combination with eutrophication and land use change is expected to exacerbate the duration, frequency, and size of cyanobacteria blooms [5,32] partially because of the anticipated increases in temperature and drought (leading to more stagnant water bodies). A report describing the specific impacts of climate change in Colorado predicted (with high confidence) increases in temperature and more frequent and intense droughts, but impacts involving cyanobacteria were not discussed [33]. Toxin-producing cyanobacteria are already a common occurrence in Colorado water bodies during warm months and these predicted climate trends suggest that blooms will become more frequent in the future. Recent work in Europe showed that climate change impacts cool lakes (high latitude) and warm lakes (low latitude) differently and found that cyanobacteria blooms in the high latitude lakes produced more toxin [34]. In some contexts, ecosystems in high latitude and high altitude environments share similar characteristics e.g., cold temperatures and short growing seasons, but It is not clear if results from high latitude European lakes can be applied to high altitude Colorado lakes.

There are approximately 1,533 public lakes in Colorado with over 10 surface acres in size [35]. Cyanobacteria naturally occur in Colorado waterways and only become a public health threat when they produce toxins. Only some of the water bodies in Colorado are routinely monitored for the presence of cyanobacteria and their toxins. These data, from the Colorado Department of Public Health & Environment (CDPHE) from 2014 to present, show that toxin-producing cyanobacteria blooms have been documented across a wide swath of the state. The CyAN project provides satellite-derived cyanobacteria concentrations for water bodies in the continental United States, but this project has only been available since 2020 and is limited to larger water bodies [25,26]. Cyanobacteria blooms have been documented in Colorado reservoirs [36,37] and in the artificial Sheldon Lake [38]. Earlier in 2022, the Pikeview Reservoir was removed as a municipal water source due to cyanobacteria [39]. While municipal water treatment plants have some strategies to remove cyanobacteria and their toxins, home water filtration units typically are not able to [40]. In 2023 and 2024, several cyanobacteria blooms were documented in multiple water bodies in Colorado, some of which resulted in closure to recreational use [41–43]. The exact history of the prevalence of toxic cyanobacteria in Colorado waterways is not well known due to a lack of long-term, routine monitoring. Recently, however, advances in satellite monitoring combined with *in situ* measurements make an investigation into public health effects plausible.

## Materials & Methods

### Identification of High Risk Water Bodies and Zip Codes

**Figure 1:**
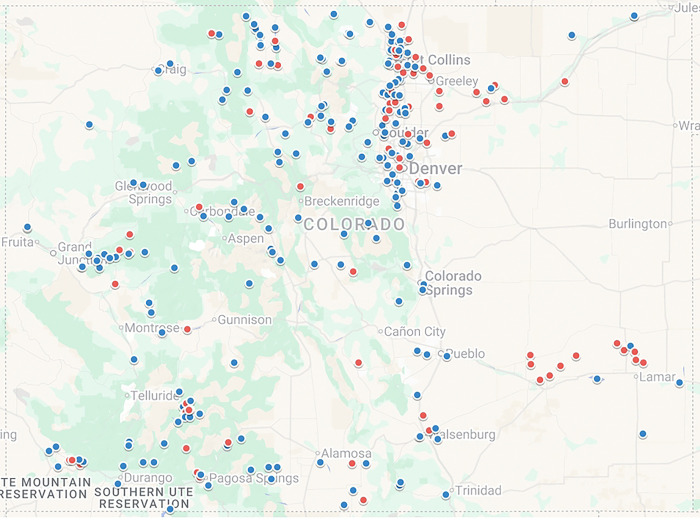
Map of Colorado Water Bodies Examined in this Study. Water bodies were labeled as being low risk for cyanobacteria toxin exposure (blue) or high risk for cyanobacteria toxin exposure (red) based on data from CyAN and CDPHE. Three low-risk water bodies are not on the map because we were unable to get accurate coordinates. Figure made using Google MyMaps.

We requested data from the CyAN project by manually searching for and choosing water bodies in Colorado using the CyAN web application and making a data request [44]. CyAN data were accessed 25 May 2023. Data from the CDPHE were gathered from their website the same month [45]. Combining these two partially overlapping data sources gave information for 276 water bodies (Fig. 1; Supp Data 1). A water body was identified as having chronic cyanobacteria blooms if: cyanobacteria cell concentrations reached 10^6^ cells per mL at least once, cyanobacteria cells were observed for at least three years, and, if tested, toxins were found. Seventy-seven water bodies met these criteria. Maps of zip codes and aerial imagery were used to identify the zip codes that had at least 95% of its residential population within ~5 km of these 77 water bodies (Fig. 1). These were the “high risk” zip codes. “Low risk” zip codes were identified as having the vast majority of its residential population either not near any water body or only near moving water (Supp Data 2).

### Identification of COVID Patients

The N3C Enclave is a secure platform through which harmonized clinical data provided by contributing members of N3C are stored [7]. The Enclave includes demographic and clinical characteristics of patients who have been tested for or diagnosed with COVID-19, and further information about the strategies and outcomes of treatments for those suspected or confirmed to have the virus. The N3C Enclave is available for public research use. To access data including that used in this manuscript, institutions must have a signed Data Use Agreement executed with the U.S. National Center for Advancing Translational Sciences (NCATS) and their investigators must complete mandatory training and submit a Data Use Request (DUR) to N3C. To request N3C data access, researchers must follow instructions at https://covid.cd2h.org/onboarding. This project can be replicated within the N3C Enclave by fully onboarded, trained users with an approved DUR. More than 4,000 researchers currently have access to data in N3C; together they represent more than 300 US research institutions. Code used in this analysis and all concept sets are available at [46]. Reviewers can confidentially request access by these same means.

The N3C data transfer to NCATS is performed under a Johns Hopkins University Reliance Protocol # IRB00249128 or individual site agreements with NIH. The work presented here was performed under a University of Colorado Anschutz Medical Campus (23–1860) and University of North Carolina Chapel Hill (24–2680) IRB protocols. A waiver of informed consent was approved by the NIH Intramural IRB for storing, operational management, and maintaining data within the N3C Enclave. This was done under the HHS regulations at 45 CFR 46. The need for consent was waived by the ethics committee. This study did not include minors.

**Figure 2:**
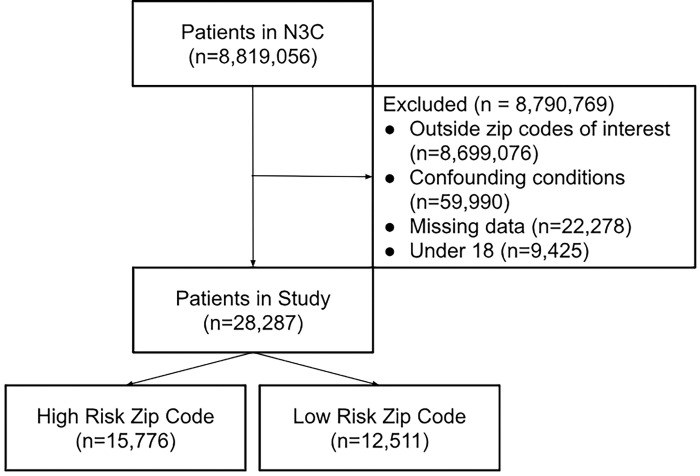
Consort Flow Diagram. This study started with over 8 million patients in N3C and Identified approximately 28K patients that met the study criteria.

N3C hosts a very large collection of de-identified electronic health records in the OMOP Common Data Model (CDM), with data available beginning 1/1/2018. Analyses were performed on N3C Level 3 data from release v185 (10/10/2024) representing over 23.3 million patients from 85 pseudo-anonymous data contributing sites across the United States [7,47]. This data set contained two HIPAA identifiers; thus, some individuals might be identifiable after data collection with extra work, which was forbidden by the data use agreement. We filtered to include only patients associated with a defined high-risk or low-risk zip code, effectively restricting to 45 data contributors. We considered only COVID-19 positive patients: those with a positive PCR test, Antigen test, or diagnosis. The first instance of these was used to identify primary COVID-19 infection date and subsequent severity as either hospitalization, Emergency Department presentation (with accompanying positive test or diagnosis), or death within 60 days of primary diagnosis. Data were filtered to include only patients associated with defined high-risk (1) or low-risk zip codes (0), and also excluded smokers, pregnant patients, minors, and those with diabetes, obesity, heart disease, organ transplant, or stroke prior to primary COVID-19 infection (Fig. 2).

Patient covariates included vaccination status (1 or more prior to primary infection), discretized age (adult 19–65, senior >65), discretized county-level poverty rate from 2019 American Community Survey data [48] (low 0–5%, middle 5–15%, high >15%), and discretized healthcare access from the 2020 Sharecare-BUSPH Social Determinants of Health Index [49] (low 0–1.5 MDs per thousand population, reasonable 1.5–2.5, strong >2.5). To allow sufficient group sizes for analysis, race and ethnicity were included and combined into categories Latino (Hispanic or Latino ethnicity), White (race White), Black (race Black or African American), and Other (race Asian, American Indian or Alaska Native, or Native Hawaiian or Other Pacific Islander.) Patients without complete covariate data were excluded from further analyses. Supplemental table 3 describes patient group counts, ranging from <20 individuals to 5,876 individuals.

### Statistical Analysis

All statistical analyses were performed in R v4.4.2. High- and low-risk patients were matched with ATT as the estimand, using exact matching on age category, race, and sex using MatchIt v4.6.0 [50]. Binary COVID-19 severity was modeled with generalized linear logistic regression, weighted with with stratum propensity score weights using glm() in R, using formula severity_type ~ cyano_risk * (vaccine_status + poverty_category + access_category). Odds Ratios (ORs) were computed using reported model coefficients.

## Results

This study was performed using data from ~28k patients and was well balanced across sex, age, and high vs low cyanobacteria toxin risk (Table 2). There were many more patients who did not have a severe COVID-19 outcome than did.

**Figure 3:**
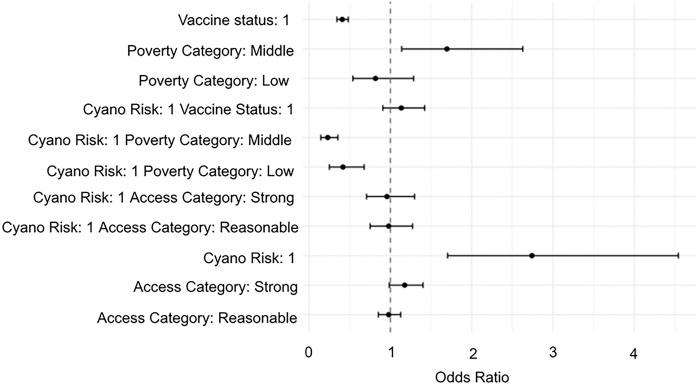
Forest Plot of Odds Ratio from GLM. Patients living in zip codes identified as high risk for chronic exposure to low levels of cyanobacteria toxin were more likely to have a severe COVID-19 outcome. There are interactions that reduce COVID-19 severity for patients living in high risk zip codes that are also wealthy or middle-class.

Compared to the baseline group (low risk, unvaccinated, high poverty, low care access), patients in high risk areas were significantly more likely to experience a severe COVID-19 outcome (OR 2.7, p<0.0001) (Supp Data 4, Fig 3). Model coefficients also suggest that vaccinated patients were less likely to have a severe COVID outcome, which is supported by prior knowledge [51,52]. There also appear to be interactions that reduce COVID severity for wealthy and middle-class patients living in high cyanobacteria risk areas [52,53].

## Discussion

The World Health Organization estimated that 3.4 million people died during the COVID-19 pandemic [54] and, as of the time of writing, thousands per month continue to die, mostly in the USA [55]. Factors that impact COVID severity thus remain impactful areas of research. The Centers for Disease Control identified several underlying medical conditions (e.g., obesity and chronic kidney disease) and demographic factors (e.g., age and race) that increase the likelihood of a severe COVID outcome [56]. Additionally, several ACE2 receptor gene variants in humans have been identified as impacting COVID susceptibility, and prevalence of these variants differs by ethnic group [57–59]. There is indirect evidence of some environmental exposures increasing the likelihood of a severe COVID outcome, such as air pollution (particulate matter, NO2, ozone), heavy metals, and endocrine disruptors [60]. Direct evidence of the impact and specific mechanism of action of a chemical or pollutant on COVID severity has not been firmly established.

Chronic exposure to low-levels of aerosolized cyanobacteria toxins could increase likelihood of a severe COVID outcome by impairing respiratory function. It is well established that comorbidities impairing respiratory function, such as asthma and chronic lung disease, can make a patient more likely to have a severe COVID-19 outcome [61–63]. While our results suggest this mechanism of action, this study did not look for direct evidence of impaired lung function in areas of high risk of exposure to cyanobacteria toxins. The complete effects of spending significant amounts of time near water bodies with chronic cyanobacteria blooms is not yet clear.

This work examines an understudied environmental exposure, chronic low-level exposure to cyanobacteria toxins, and its impact on public health. These findings suggest that in addition to the direct impact of the toxins on organ and tissue function, chronic, low-levels of cyanobacteria toxins can increase an individual’s susceptibility to some communicable disease. Further study is needed to see if these results are applicable to a larger population and to identify the specific mechanism by which proximity to recurring cyanobacteria blooms leads to more severe COVID-19 outcomes.

## Supplementary Material

Supporting Information Captions

S1 Data: Colorado Waterbody and Cyanobacteria Data Summary

S2 Data: High Risk and Low Risk Zip Codes Used in This Study

S3 Data: Patient Demographic Summary

S4 Data: GLM Results and Odds Ratios

## Figures and Tables

**Table 1.** **Cyanobacteria Toxins and Their Mode of Action.** Recreated from [6]

Toxin	Mode of Action	Main Effect
Microcystins	Inhibits protein phosphatase	Liver failure and hepatic hemorrhage
Nodularins	Inhibits protein phosphatase	Liver failure and hepatic hemorrhage
Cylindrospermopsins	Inhibits protein synthesis	Liver and kidney failure
Anatoxin-a	Binds to nicotinic acetylcholine receptors	Muscular paralysis
Anatoxin-a(s)	Inhibits acetylcholinesterase	Muscular weakness, dyspnea, and convulsions
Saxitoxins	Binds to sodium channels	Ataxia, convulsions, and paralysis
BMAA	Binds to glutamate receptors	Neurodegenerative syndrome
Aplysiatoxins	Activates protein kinase C	Tumor promotion and skin irritation
Lyngbyatoxins	Activates protein kinase C	Tumor promotion and skin irritation

**Table 2:** Patient Demographics

Number	28,287
Male / Female	12,236 / 16,051
Age	45.8 +/− 17.9
Severe COVID / Not severe COVID	3,570 / 24,717
High Risk / Low Risk	15,776 / 12,511
